# Exploring novel semi-inner product reproducing Kernels in Banach space for robust Kernel methods

**DOI:** 10.1371/journal.pone.0340686

**Published:** 2026-01-16

**Authors:** Yi Ding, Ying Zhao, Yan Pei

**Affiliations:** 1 Graduate School of Computer Science and Engineering, University of Aizu, Aizu-wakamatsu, Fukushima, Japan; 2 Computer Science Division, University of Aizu, Aizu-wakamatsu, Fukushima, Japan; Universidad CEU Cardenal Herrera - Campus Elche, SPAIN

## Abstract

Kernel methods are widely applied across various domains; however, structural limitations of reproducing kernels in Hilbert spaces pose significant challenges. Many challenges inherent to Hilbert spaces can be effectively addressed within the framework of Banach spaces. In this work, we define the semi-inner product reproducing kernel Banach space and its reproducing kernels using semi-inner product and bilinear mapping, supported by rigorous proofs. Specific forms of semi-inner product reproducing kernels are derived within the theoretical framework of the semi-inner product reproducing kernel Banach space. This constitutes the core originality of our work and represents its primary contribution. Through illustrative experiments, we validate the effectiveness of semi-inner product reproducing kernels and demonstrate their superior performance compared to polynomial reproducing kernels.

## 1 Introduction

The reproducing kernel theory originated from integral theory. During its initial development, the kernel was regarded as the continuous kernel of a positive definite integral operator. This theory was introduced by Mecer and termed the positive definite kernel [1]. The theoretical framework of reproducing kernel Hilbert space (RKHS) was proposed in the 1930s. Bergman [2] investigated the boundary value problem of differential equations as shown in Eq (1). In this context, *a*(*x*,*y*), *b*(*x*,*y*), $c(x,y)\in {\mathbb{C}}^{2}(ℰ)$, that is, *a*(*x*,*y*), *b*(*x*,*y*), and *c*(*x*,*y*) are bivariate square-integrable functions on the interval ℰ. The concept and formulation of the reproducing kernel were given for the first time. Aronszajn summarized the previous work, developed the reproducing kernel theory incorporating the Bergman kernel, and streamlined the proof process [3].

$$\frac{\partial^{2}u}{\partial x^{2}}+\frac{\partial^{2}u}{\partial y^{2}}+a(x,y)\frac{\partial u}{\partial x}+b(x,y)\frac{\partial u}{\partial y}+c(x,y)u=0.$$ (1)

Reproducing kernel functions are widely utilized in mathematics and computer science. In mathematical fields, such as functional analysis, numerical analysis, and partial differential equations, kernel functions are prevalent. In computer science, reproducing kernel functions are primarily applied in kernel methods, which play a vital role in machine learning [4], pattern recognition, and statistics. In the field of kernel methods, there have been several works applying reproducing kernel functions. In support vector machines (SVM), kernel functions are utilized to transform data from lower-dimensional feature space to a high-dimensional feature space to find better linear segmentation surfaces, i.e., linear decision boundaries [5–9]. Bernhard introduced kernel methods into the principal component analysis (PCA), performing PCA in the high-dimensional space mapped by kernel functions to reduce data dimensionality [10]. He extended reproducing kernels into the k-means clustering algorithm, enabling the algorithm to handle clustering problems with more complex shapes [11]. Parzen utilized kernel functions to estimate probability density functions, which are extensively employed in data analysis and pattern recognition [12]. Mika employed kernel functions to enhance the linear discriminant analysis (LDA) algorithm, allowing it to manage nonlinear classification problems [13].

It is well-known that the Hilbert space ℋ and its dual space ${ℋ}^{*}$ are isometrically isomorphic, meaning they are essentially equivalent in this context. Therefore, the inner product $\langle \cdot ,\;\cdot \rangle_{ℋ}$ defined on ℋ can also be interpreted as being defined on both ℋ and its dual space ${ℋ}^{*}$. This property enables the classical reproducing properties to be described in terms of dual spaces and dual bilinear forms, and serves as a foundation for extending the reproducing properties from Hilbert spaces to Banach spaces.

**Fig 1:**
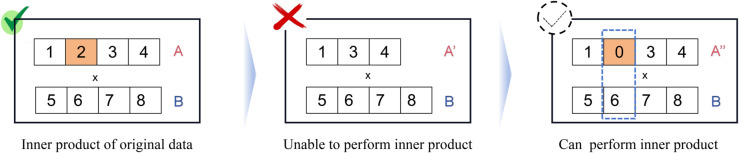
A and B are two vectors of the same dimension, and these two can be the inner product, but when A loses the data of one dimension and becomes A’, A’ and B cannot perform inner product, as A’ and B are vectors of two different dimensions. To solve the problem, the common way is to use the method of zeroing in the location of the lost data to get the A” vector, making it possible to perform an inner product operation. However, this zero-filling method assumes that the corresponding component of vector B in the missing dimension is zero, rendering the contribution of vector B in that dimension meaningless during the inner product operation.

Compared with Hilbert space, Banach space offerss distinct advantages when solving certain classes of problems. First, any two Hilbert spaces of the same dimension are isometrically isomorphic, meaning they can essentially be considered as the same space. In contrast, two Banach spaces of the same dimension, p≠q∈[1,∞], *L*^*p*^[0,1] and *L*^*q*^[0,1], are not isomorphic, i.e. two different spaces [14]. Consequently, for a given dimension, Banach spaces exhibit richer geometric structure than Hilbert spaces. This structural advantage can potentially improve the performance of kernel-based machine learning algorithms. Second, Hilbert spaces lack the flexibility to accommodate the intrinsic geometric structure present in many real-world datasets [15]. Consequently, traditional machine learning algorithms based on RKHS may fail to process such data. In contrast, machine learning algorithms based on Banach spaces can address this issue, as data with intrinsic structure can be embedded into Banach spaces. Third, during data processing, algorithms frequently apply a large number of inner product operations. Certain dimensions of the data may be lost during reading and processing. In real-world scenarios, the number of dimensions does not always match among the datasets, preventing inner product operations with other data. Currently, missing data dimensions are commonly filled with zeros [16] to ensure consistent dimensionality, thereby enabling kernel methods based on inner product operations. As shown in Fig 1, when vector *A* loses one dimension of data and becomes $A^{\prime }$, the inner product operation cannot be performed. The missing data is filled with zeros to obtain vector $A^{\prime \prime }$, enabling the inner product operation to be performed again. However, the zero-filling method assumes that the missing data component in a given dimension is zero, making the corresponding values of other data meaningless in the inner product operation. In other words, if a dimension is missing from a piece of data, it is equivalent to losing that dimension across all data during inner product operations. A number of other padding methods have been adopted to make the inner product computable, such as mean imputation [17] and other statistical approaches [18]. Although these methods facilitate the calculation of the inner product, they can result in significant deviations. If an alternative operation can effectively substitute the inner product in data processing, more accurate and reasonable results for the designed machine learning algorithms may be achieved. In light of the above considerations, we aim to identify a function that can replace the inner product operation when generating reproducing kernel functions. This objective forms the core motivation for our proposal and the present work.

In this paper, we first start with the theoretical foundation and provide a complete proof of the reproducing kernel Banach space theory. Based on the relationship between the reproducing kernel Hilbert space and the reproducing kernel, we present the theoretical form of the reproducing kernel in the Banach spaces. We propose the introduction of a bilinear mapping similar to the inner product in the Banach space and introduce a semi-inner product in the Banach space. Due to certain structural issues within the space, we add additional properties to the Banach space, thereby establishing the theorem of the semi-inner product reproducing kernel Banach space and the reproducing kernel. Subsequently, we explore practical applications by providing several specific forms of the semi-inner product reproducing kernel that are applicable. Through motivating examples, we verify the effectiveness of the semi-inner product reproducing kernel in algorithmic applications.

Following this introduction, Sect 2 briefly discusses the relationship between the reproducing kernel Hilbert space and the reproducing kernel, as well as the process of constructing the reproducing kernel. In Sect 3, we provide a complete proof of the necessary and sufficient conditions for the existence of a reproducing kernel Banach space and its reproducing kernel. We also justify the introduction of a semi-inner product in Banach spaces and discuss its additional properties. Finally, we derive the semi-inner product reproducing kernel. In Sect 4, specific forms of semi-inner product reproducing kernels are presented. Subsequently, we validate the effectiveness of these kernels through motivational examples and compare them with polynomial reproducing kernels in Sect 5. Sect 6 summarizes the contributions of this study and discusses key issues to outline directions for further research.

## 2 Review of the reproducing Kernel Hilbert space

Before giving the theory and proof of the reproducing kernel Banach space, we have reviewed the reproducing kernel theory in Hilbert space. This review addresses three key points as follows.

- The reproducing kernel Hilbert space (RKHS) is defined by the evaluation function.
- The necessary conditions for the existence of reproducing kernels.
- The positive definiteness property that enables kernel construction. The construction is implemented using a method provided by the Moore-Aronszajn theorem. A function must be positive definite to serve as a reproducing kernel, ensuring that it can define an inner product and generate the corresponding RKHS using the Moore-Aronszajn theorem.

### 2.1 Reproducing Kernel Hilbert space

**Definition:** Let where *X* is a set, RKHS ℋ is a Hilbert space of functions on *X*, if the evaluation functional $\delta_{x}$ is a continuous linear functional.

Let K:X×X→ℂ be a reproducing kernel on *X*. Then there exists a unique RKHS ℋ satisfying:

1. The Membership Property: K(x,·)∈ℋ, for each x∈X;
2. The Reproducing Property: For any f∈ℋ,
  $$\delta_{x}(f)=f(x)=\langle f,K(x,\cdot )\rangle_{ℋ},$$ (2)
  $\langle \cdot ,\cdot \rangle_{ℋ}$ denotes the inner product on ℋ;
3. The Inner Product Between Kernel Functions Property: Let f:=K(y,·) for x,y∈X, then we can obtain,
  $$f(x)=K(y,x)=\langle K(y,\cdot ),K(x,\cdot )\rangle_{ℋ}.$$ (3)

A RKHS is a Hilbert space of functions in which the evaluation of a function at any point can be represented as an inner product. Specifically, given a positive definite kernel function *K*(*x*,*y*), the RKHS ℋ associated with *K* consists of functions *f* such that for all x∈X, the reproducing property of Eq (2) holds. This reproducing property guarantees that pointwise evaluation is a continuous linear functional, a critical feature that distinguishes RKHS from general Hilbert spaces. Moreover, each RKHS corresponds uniquely to a kernel function, and the inner product structure it inherits from Hilbert spaces facilitates rigorous mathematical analysis and optimization.

### 2.2 Moore-Aronszajn theorem

**Theorem 1** (*Moore-Aronszajn Theorem*). *Let* K:X×X→ℝ *be positive definite. There exists a unique RKHS* $ℋ\subset {\mathbb{R}}^{X}$ *associated with the reproducing kernel K. Furthermore, if space* ${ℋ}_{0}=\{K(\cdot ,x)|x\in X\}$ *is equipped with the inner product*


$$\langle f,g\rangle_{{ℋ}_{0}}=\sum_{i=1}^{n}\sum_{j=1}^{m}\alpha_{i}\beta_{j}K\left(y_{j},x_{i}\right)$$

*where* $f=\sum_{i=1}^{n}\alpha_{i}K\left(\cdot ,x_{i}\right)$ *and* $g=\sum_{j=1}^{m}\beta_{j}K\left(\cdot ,y_{j}\right)$*, then* ${ℋ}_{0}$ *constitutes a valid RKHS.*

We can derive the following conclusions from the proof of the RKHS. First, in the process of proving the reproducing kernel, since the Hilbert space ℋ is self-conjugate, we can introduce the isometric isomorphic mapping $I:{ℋ}^{*}\to ℋ$. This allows us to replace the elements in ${ℋ}^{*}$ with the elements in ℋ to complete the proof. In case of reproducing kernel Banach space (RKBS) ℬ, we retain certain properties of ℋ. We assume that RKBS ℬ is reflexive, meaning that $\left({ℬ}^{*}\right){\;\;}^{*}=ℬ$. Second, in RKHS, since the Riesz representation theorem holds, *f*(*x*) can be expressed as an inner product. The inner product can be viewed as a bilinear map between $ℋ\;\times \;{ℋ}^{*}$. It is called a bilinear map because ∀a,b∈ℂ,f,g∈ℋ, and $f^{*},g^{*}\in {ℋ}^{*}$ the following holds:


$${\left\langle af+bg,f^{*}\right\rangle }_{ℋ}=a{\left\langle f,f^{*}\right\rangle }_{ℋ}+b{\left\langle g,f^{*}\right\rangle }_{ℋ}$$

and


$${\left\langle f,af^{*}+bg^{*}\right\rangle }_{ℋ}=a{\left\langle f,f^{*}\right\rangle }_{ℋ}+b{\left\langle f,g^{*}\right\rangle }_{ℋ}.$$

Third, the reproducing kernel is intrinsically a bivariate function that becomes a univariate function when one variable is fixed. The Moore-Aronszajn theorem utilizes the space spanned by this univariate function to define the RKHS, thereby providing a method for constructing RKHS. This inspires the construction of RKBS, and we will adopt a similar strategy to design algorithms within the RKBS framework.

## 3 Theorem and proof of the reproducing Kernel Banach space

When generalizing the reproducing kernel to a Banach space, a bilinear form analogous to Eq (2) in the Banach space must be introduced. Specifically, we aim to extend the Riesz representation theorem to the Banach space. To achieve this, we define the bilinear form of $ℬ\;\times \;{ℬ}^{*}$ on the normed vector space ℬ

$${\left(x,y^{*}\right)}_{ℬ}:=y^{*}(x),\;x\in ℬ,y^{*}\in {ℬ}^{*}.$$ (4)

If the Riesz Representation Theorem holds on RKBS, that is to say, ∀x∈X, because $\delta_{x}$ is a bounded linear functional on ℬ, there $\exists y_{x}\in {ℬ}^{*}$ so that

$$\delta_{x}(f)=f(x)={\left(f,y_{x}\right)}_{ℬ}.$$ (5)

Based on the definitions and properties of RKHS, we formally introduce the corresponding definitions and fundamental properties for RKBS.

**Definition:** A Reproducing Kernel Banach space (RKBS) ℬ is a Banach space of functions defined on *X* in which the evaluation functional $\delta_{x}$ is a continuous linear functional [19].

**Property:** Let K:X×X→ℂ be a reproducing kernel on *X*. Then there exists a unique RKBS ℬ with dual space ${ℬ}^{*}$ satisfying the following conditions:

1. The Function Property: $K(\cdot ,x)\in {ℬ}^{*}$ for all x∈X;
2. the Reproducing Property: For any f∈ℬ,
  $$f(x)=(f,K(\cdot ,x){)}_{ℬ}$$
  where (·,·) denotes the dual bilinear pairing on $ℬ\times {ℬ}^{*}$;
3. The Inner Product Between Kernel Functions Property: For any x,y∈X, let f:=K(y,·),
  $$f(x)=K(y,x)=(K(y,\cdot ),K(\cdot ,x){)}_{ℬ}.$$

If the Riesz representation theorem holds on a Banach space, Theorem 2 can be rigorously derived.

### 3.1 Densify theorem of the reproducing Kernel Banach space

The densification theorem in RKBS theory establishes that the linear span of reproducing kernels forms a dense subset of the entire space. Here, density implies that every element in the RKBS admits arbitrarily close approximation (with respect to the space’s norm) by finite linear combinations of reproducing kernels. This fundamental result holds significant importance in both functional analysis and kernel-based machine learning, extending the classical density property of RKHS to the more general and structurally complex setting of Banach spaces.

#### 3.1.1 Theorem and proof summary.

**Theorem 2.** *Let* ℬ *be an RKBS on X, defined by*


$$ℬ:=\left\{f|f(x)={\left(u,\Phi^{*}(x)\right)}_{\Omega },x\in X,u\in \Omega \right\},\;\text{and norm}{\left\|f\right\|}_{ℬ}:=\|u{\|}_{\Omega },$$

*and let its conjugate space* ${ℬ}^{*}$, given by


$${ℬ}^{*}:=\left\{g|g(x)={\left(\Phi (x),u^{*}\right)}_{\Omega },x\in X,u^{*}\in \Omega^{*}\right\},\;\text{and norm}{\left\|g\right\|}_{{ℬ}^{*}}:={\left\|u^{*}\right\|}_{\Omega^{*}},$$

*where* Ω *and* $\Omega^{*}$ *are reflexive Banach spaces with* $\Omega^{*}$ *being the dual of* Ω*, and there exist two maps* Φ:X→Ω *and* $\Phi^{*}:X\to \Omega^{*}$ *such that*

$$\text{span }\Phi (X)\text{ is dense in }\Omega ,\text{ span }\Phi^{*}(X)\text{ is dense in }\Omega^{*}$$ (6)

*The bilinear form between* ℬ *and* ${ℬ}^{*}$ *is defined by*

$${\left(f(\cdot ),g(\cdot )\right)}_{ℬ}:={\left(u,u^{*}\right)}_{\Omega }$$ (7)

*A function* K:X×X→ℂ *is the reproducing kernel of an RKBS on X if and only if the reproducing kernel K is expressed by Eq* (8)

$$K(x,y):={\left(\Phi (x),\Phi^{*}(y)\right)}_{\Omega },\;x,y\in X$$ (8)

*and* $\Phi :X\to \Omega ,\Phi^{*}:X\to \Omega^{*}$ *satisfies the conditions of Eq* (6).

The proof of the theorem is divided into two parts, i.e., sufficiency and necessity. The proof of sufficiency consists of three steps. First, we prove that ℬ is a Banach space. Since the construction of ${ℬ}^{*}$ is analogous to that of ℬ, and ${ℬ}^{*}$ is known to be a Banach space, we begin by verifying the density condition. Next, we demonstrate that both ℬ and ${ℬ}^{*}$ are RKBS over *X*. Specifically, we prove that the evaluation function $\delta_{x}f$ is continuous on both ℬ and ${ℬ}^{*}$. This is established using the Cauchy–Schwarz inequality. Finally, we use a constructive approach to verify that the reproducing kernel expression given in the theorem satisfies the criteria for being a reproducing kernel.

The proof of necessity also consists of three steps. First, we prove the uniqueness of the reproducing kernel by contradiction. Then, using a constructive method, we derive the explicit expression of the kernel given in the theorem. Finally, we prove the dense condition. We use inference from the Hahn-Banach theorem and a proof by contradiction. By assuming the density condition does not hold, we derive a contradiction. Therefore, the assumption is invalid, and the dense condition is confirmed. Taken together, these steps complete the proof of the theorem.

#### 3.1.2 Proof of sufficiency and necessity in the theorem.

**Proof of sufficiency.** Let u∈Ω and assume that $f(x)={\left(u,\Phi^{*}(x)\right)}_{\Omega }=0$, ∀x∈X. Because $\mathrm{span}\Phi^{*}(X)$ is dense in $\Omega^{*}$, we have $\overset{―}{\mathrm{span}}\Phi^{*}(X)=\Omega^{*}$. Therefore, ${\left(u,u^{*}\right)}_{\Omega }=0$, $\forall u^{*}\in \Omega^{*}$, implying that *u* = 0. Conversely, if *u* = 0, then $f(x)={\left(u,\Phi^{*}(x)\right)}_{\Omega }=0$, ∀x∈X, which is obvious. This demonstrates that the representation of the function $\left(u,\Phi^{*}(\cdot )\right)$ in ℬ is unique. In other words, we can use *u* to represent $\left(u,\Phi^{*}(\cdot )\right)$, i.e., each function in ℬ can be uniquely represented by the element u∈Ω.

Based on this one-to-one mapping relationship, we define the norm on the space ℬ as follows: ${\left\|{\left(u,\Phi^{*}(\cdot )\right)}_{\Omega }\right\|}_{ℬ}:=\|u{\|}_{\Omega }$. Since Ω is a Banach space, ℬ endowed with this norm consequently forms a Banach space. Similarly, $\tilde{ℬ}:=\left\{g(x)={\left(\Phi (x),u^{*}\right)}_{\Omega }|x\in X,u^{*}\in \Omega^{*}\right\}$ is a Banach space. Define the bilinear form on $ℬ\;\times \;\tilde{ℬ}$ as ${\left(f(\cdot ),g(\cdot )\right)}_{ℬ}:={\left(u,u^{*}\right)}_{\Omega }.$ Clearly, $\forall u\in \Omega ,u^{*}\in \Omega^{*}$ that


$$|\left(f(\cdot ),g(\cdot )\right)|=\left|\left({\left(u,\Phi^{*}(\cdot )\right)}_{\Omega },{\left(\Phi (\cdot ),u^{*}\right)}_{\Omega }\right)\right|\;\leq \|u{\|}_{\Omega }{\left\|u^{*}\right\|}_{\Omega^{*}}\;={\left\|{\left(u,\Phi^{*}(\cdot )\right)}_{\Omega }\right\|}_{ℬ}{\left\|{\left(\Phi (\cdot ),u^{*}\right)}_{\Omega }\right\|}_{\tilde{ℬ}}.$$

Consequently, every function in $\tilde{ℬ}$ is a bounded linear functional on ℬ. This is because the linear mapping u→f(·) is isometric from Ω to ℬ. Therefore, $\tilde{ℬ}$ contains all bounded linear functionals on ℬ, implying that $\tilde{ℬ}={ℬ}^{*}$. Since Ω is reflexive, ℬ is the conjugate space of ${ℬ}^{*}$, implying that both ℬ and ${ℬ}^{*}$ are reflexive. In addition, ∀x∈X,


$$|\delta_{x}f|=|f(x)|=\left|{\left(u,\Phi^{*}(x)\right)}_{\Omega }\right|\leq \|u{\|}_{\Omega }{\left\|\Phi^{*}(x)\right\|}_{\Omega^{*}}=\|f{\|}_{ℬ}{\left\|\Phi^{*}(x)\right\|}_{\Omega^{*}}.$$

Similarly, it can be proved that the evaluation functional $\delta_{x}$ is bounded on ℬ. Therefore, ℬ is the RKBS over *X*. Next, we prove the existence of the reproducing kernel: ∀x∈X, K(x,·)∈ℬ and $K(\cdot ,x)\in {ℬ}^{*}$.


$$\delta_{x}f=f(x)={\left(u,\Phi^{*}(x)\right)}_{\Omega }={\left({\left(u,\Phi^{*}(\cdot )\right)}_{\Omega },{\left(\Phi (\cdot ),\Phi^{*}(x)\right)}_{\Omega }\right)}_{ℬ}=(f,K(\cdot ,x){)}_{ℬ}$$

$$\delta_{x}f^{*}=f^{*}(x)={\left(\Phi (x),u^{*}\right)}_{\Omega }={\left({\left(\Phi (x),\Phi^{*}(\cdot )\right)}_{\Omega },{\left(\Phi (\cdot ),u^{*}\right)}_{\Omega }\right)}_{ℬ}={\left(K(x,\cdot ),f^{*}\right)}_{ℬ}$$

This demonstrates that *K* is the reproducing kernel of ℬ. Therefore, the sufficiency part is proved.

**Proof of necessity.** Since ℬ is an RKBS over *X* and *K* is its reproducing kernel, $\delta_{x}$ is bounded. In other words, $\exists g_{x}\in {ℬ}^{*}$ such that ∀x∈X


$$\delta_{x}f=f(x)={\left(f,g_{x}\right)}_{ℬ},\;\forall f\in ℬ.$$

Let $\tilde{k}:X\times X\to \mathbb{C}$, assume that $\tilde{K}(x,y):=g_{x}(y)$, such that $\tilde{K}(x,\cdot )\in {ℬ}^{*}$, ∀x∈X. Then,

$$f(x)=(f,\tilde{K}(x,\cdot ){)}_{ℬ},\;f\in ℬ.$$ (9)

Suppose instead there exists another function $\tilde{G}:X\times X\to \mathbb{C}$ satisfying $\{\tilde{G}(x,\cdot )|x\in X\}\subseteq {ℬ}^{*}$, and


$$\delta_{x}f=f(x)=(f,\tilde{G}(x,\cdot ){)}_{ℬ},\;f\in ℬ.$$

Then $(f,\tilde{K}(x,\cdot )-\tilde{G}(x,\cdot ){)}_{ℬ}=0,\forall f\in ℬ$, $∴\tilde{K}(x,\cdot )-\tilde{G}(x,\cdot )=0$. Since $\tilde{K}(x,\cdot )$, $\tilde{G}(x,\cdot )\in {ℬ}^{*}$, so, for all y∈X, we have


$$\tilde{K}(x,y)-\tilde{G}(x,y)=0.$$

Therefore, the function $\tilde{K}$ is exactly the same as the function $\tilde{G}$, in other words, $\tilde{K}=\tilde{G}$. Similarly, there exists a unique function K:X×X→ℂ such that K(y,·)∈ℬ,y∈X and $f^{*}(y)={\left(K(y,\cdot ),f^{*}\right)}_{ℬ}$.

Let f:=K(y,·), and $f^{*}:=\tilde{K}(x,\cdot )$, then

$$f(x)=K(y,x)=(K(y,\cdot ),\tilde{K}(x,\cdot ){)}_{ℬ},$$ (10)

and

$$f^{*}(y)=\tilde{K}(x,y)=(K(y,\cdot ),\tilde{K}(x,\cdot ){)}_{ℬ}.$$ (11)

We obtain that $\tilde{K}(x,y)=K(y,x),x,y\in X$, therefore, $\tilde{K}(x,\cdot )=K(\cdot ,x)$, K(x,y)=(k(x,·),k(·,y)).

Let Φ(x):=K(x,·), $\Phi^{*}(x):=K(\cdot ,x)$, then $K(x,y)=(\Phi (x),\Phi^{*}(x))$. Let $\Omega :=ℬ,\;\Omega^{*}:={ℬ}^{*}$, and suppose that $\overset{―}{\mathrm{span}}\Phi (X)\neq \Omega$. By the Hahn-Banach theorem, $\exists f^{*}\in {ℬ}^{*}$,


$$f^{*}(x)={\left(\Phi (x),f^{*}\right)}_{ℬ}=0,\forall x\in X,\|f^{*}\|=1$$

However, the above formula implies that $f^{*}(x)=0$, ∀x∈X, that is, $f^{*}=0$. This contradicts the above formula. Therefore, $\overset{―}{\mathrm{span}}\Phi (X)=\Omega$. Similarly, $\overset{―}{\mathrm{span}}\Phi^{*}(X)=\Omega^{*}$, and the proof is complete.

### 3.2 Construction of the reproducing Kernel Banach space with properties of semi-inner product, Fréchet differentiable and convex

There are three primary properties of RKBS that should be defined. These properties indicate that a Banach space is being constructed which not only possesses a reproducing kernel structure but also incorporates the important mathematical properties. First is the semi-inner product; it allows for the definition of a certain notion of direction and magnitude in non-Hilbert spaces. Second is the Fréchet differentiability, which enables the definition of derivatives or gradients in Banach spaces, which is beneficial for functional analysis and optimization problems in machine learning. The third is the convexity. It ensures the existence of certain minimizers and the uniqueness of the reproducing kernel. The combination of these properties provides a solid foundation for both the theoretical development and practical applications of RKBS, such as in support vector machines and functional approximation.

#### 3.2.1 Semi-inner product.

From Theorem 2, it follows that an RKBS ℬ corresponds to a unique reproducing kernel. However, a single reproducing kernel may correspond to multiple RKBSs. For example, consider the kernel function $k(x,y)=x_{1}^{2}+x_{2}^{2}+y_{1}^{2}+y_{2}^{2}+2x_{1}x_{2}y_{1}y_{2}=(x,y){\;\;}^{2}$, where $x,y\in {\mathbb{R}}^{2}$. Two distinct feature mappings exist for this kernel:

- First feature mapping: $\Phi_{1}(x)=[x_{1}^{2},x_{2}^{2},x_{1}x_{2},x_{1}x_{2}]$, with the associated Banach space ${ℬ}_{1}={\mathbb{R}}^{4}$.
- Second feature mapping: $\Phi_{2}(x)=[x_{1}^{2},x_{2}^{2},\sqrt{2}x_{1}x_{2}]$, with the associated Banach space ${ℬ}_{2}={\mathbb{R}}^{3}$.

$k(x,y)=\left(\Phi_{1}(x),\Phi_{1}(y)\right)=\left(\Phi_{2}(x),\Phi_{2}(y)\right)$, this demonstrates how a single reproducing kernel *k* can correspond to two different RKBS (${ℬ}_{1}$ and ${ℬ}_{2}$) through distinct feature embeddings. Additionally, if *X* is a finite set, every non-zero functional *K* defined on X×X corresponds to the reproducing kernel of a certain RKBS on *X*.

In other words, the reproducing kernel of a general RKBS may be neither positive definite nor symmetric. This is due to the distinction between Banach space and Hilbert space. There is no inner product structure in Banach space. To ensure that the reproducing kernel of an RKBS possesses the same properties as that of an RKHS, we introduce the semi-inner product, as defined by Lumer [20], into the RKBS. A semi-inner product on a vector space ℬ is a function, denoted by $[\cdot ,\cdot {]}_{ℬ}$, that maps ℬ×ℬ to ℂ and satisfies the following conditions for all f,g,h∈ℬ and λ∈ℂ:

1. $[f,f{]}_{ℬ}\geq 0,[f,f{]}_{ℬ}=0$ if and only if *f* = 0,
2. $[f+g,h{]}_{ℬ}=[f,h{]}_{ℬ}+[g,h{]}_{ℬ}$,
3. $[\lambda f,g{]}_{ℬ}=\lambda [f,g{]}_{ℬ},[f,\lambda g{]}_{ℬ}=\bar{\lambda }[f,g{]}_{ℬ}$.

The semi-inner product differs from the inner product in that it does not satisfy the property of conjugate symmetry, that is, $[f,g{]}_{ℬ}\neq {\overset{―}{\left[g,f\right]}}_{ℬ}$. This results in the second variable of the semi-inner product being not additive, in other words

$$[f,g+h{]}_{B}\neq [f,g{]}_{B}+[f,h{]}_{B}.$$ (12)

We can easily prove that a semi-inner product is an inner product if and only if the second variable of the semi-inner product has additivity, that is

$$[f,g+h{]}_{B}=[f,g{]}_{B}+[f,h{]}_{B}.$$ (13)

In Lumer [20], it was demonstrated that ℬ endowed with a semi-inner product is a normed space, where the norm is defined by $\|f{\|}_{ℬ}:=[f,f{]}_{ℬ}^{1/2},f\in ℬ.$

$$\|f{\|}_{ℬ}:=[f,f{]}_{ℬ}^{1/2},f\in ℬ$$ (14)

If a vector space ℬ has a semi-inner product and the norm on ℬ is induced by Eq (14), we refer to ℬ as a semi-inner product space.

By the Cauchy-Schwarz inequality, if ℬ is a semi-inner product space, for each g∈ℬ, $f\to [f,g{]}_{ℬ}$ corresponds to a continuous linear functional on ℬ, we denote this linear functional as g^*^. From this definition, we obtain

$$[f,g{]}_{B}=g^{*}(f)={\left(f,g^{*}\right)}_{B},f,g\in B.$$ (15)

#### 3.2.2 Differentiable and convex characteristics.

In general, semi-inner products on normed vector spaces may not be unique. The differential properties of the norm ensure uniqueness. Let S(ℬ) denote the unit sphere on the normed vector space ℬ, $S(ℬ):=\left\{f:\|f{\|}_{ℬ}=1\right\}$. If the limit exists ∀f,g∈ℬ\{0}

$$\lim_{t\in \mathbb{R},t\to 0}\frac{\|f+tg{\|}_{ℬ}-\|f{\|}_{ℬ}}{t}$$ (16)

Moreover, if the limit converges uniformly on S(ℬ)×S(ℬ), then the normed vector space is called Fréchet differentiable [21].

Furthermore, we introduce the property of uniform convexityity to the vector space, which ensures the Riesz representation theoris valid valid on the semi-inner product space. The normed vector space ℬ is uniformly convex if ∀ε>0,∃δ>0 such that,

$$\forall f,g\in 𝒮(ℬ),\|f-g{\|}_{ℬ}\geq \varepsilon ,\text{then}\;\|f+g{\|}_{ℬ}\leq 2-\delta .$$ (17)

#### 3.2.3 Uniformly Fréchet differentiable Banach Space and uniformly convex.

According to [22], it was shown that a uniformly convex Banach space is reflexive. In addition, a normed vector space ℬ is uniformly Fréchet differentiable if and only if its dual space ${ℬ}^{*}$ is uniformly convex [23]. From these two properties, we obtain the following result. If ℬ is a uniformly convex and uniformly Fréchet differentiable Banach space, then the space ℬ is reflexive, meaning ${ℬ}^{**}$ is also a uniformly Fréchet differentiable and uniformly convex Banach space. Because ℬ is a Fréchet differentiable Banach space, then its dual space ${ℬ}^{*}$ is a uniformly convex Banach space. Moreover, since ${ℬ}^{**}$ is a uniformly convex Banach space and the dual space of ${ℬ}^{*}$ is ${ℬ}^{**}$, it follows that ${ℬ}^{*}$ is a uniformly Fréchet differentiable Banach space. In summary, ${ℬ}^{*}$ and ${ℬ}^{**}$ are uniformly convex and uniformly Fréchet differentiable Banach spaces.

Let ℬ be a uniformly convex and uniformly Fréchet differentiable Banach space. Then, for each $f\in {ℬ}^{*}$ there exists a unique y∈ℬ so that $f=y^{*}$, i.e., $f(x)=[x,y{]}_{ℬ},x\in ℬ$ [21]. Moreover, $\|f{\|}_{{ℬ}^{*}}=\|y{\|}_{ℬ}$. Let *X* be an input space, and let Ω be a uniformly Fréchet differentiable and uniformly convex Banach space, with $\Omega^{*}$ as the conjugate space of Ω. We define mapping Φ:X to Ω, $\Phi^{*}:X$ to $\Omega^{*}$, and $\Phi^{*}(x):=(\Phi (x)){\;\;}^{*},x\in X$. By combining the two conditions added above, from Theorem 2, we can obtain the following conclusions.

### 3.3 Dual mapping theorem of the reproducing Kernel Banach space

The Dual Mapping Theorem is a fundamental result in the theory of RKBS. It states that, under appropriate conditions, the dual space of an RKBS also forms an RKBS equipped with a corresponding dual reproducing kernel. This implies that the reproducing property is preserved not only in the original space but also in its dual, allowing any continuous linear functional to be represented via pairing with the dual kernel function. The theorem plays a crucial role in functional analysis and kernel-based machine learning, providing a theoretical foundation for extending kernel methods to more general Banach spaces, such as those based on *L*^*p*^ norms, for tasks like regression and classification.

**Theorem 3.** *Let* Φ *be a function that maps X to* Ω*, and* $\Phi^{*}$ *is a function that maps X to* $\Omega^{*}$, $\Phi^{*}(x):=(\Phi (x)){\;\;}^{*}$ *so that*

$$\text{span }\Phi (X)\text{ is dense in }\Omega ,\text{ span }\Phi^{*}(X)\text{ is dense in }\Omega^{*}.$$ (18)

*Then* ℬ *is uniformly Fréchet differentiable and uniformly convex Banach space,* $ℬ:=\left\{f_{u}|f_{u}(\cdot )=[u,\Phi (\cdot ){]}_{\Omega },u\in \Omega \right\}$ and with norm ${\left\|f\right\|}_{ℬ}:=\|u{\|}_{\Omega }$*. The semi-inner product on the space* ℬ *can be expressed as*

$${\left[f_{u}(\cdot ),f_{v}(\cdot )\right]}_{ℬ}:=[u,v{]}_{\Omega }$$ (19)

*and* ${ℬ}^{*}$ *is a uniformly Fréchet differentiable and uniformly convex Banach space,* ${ℬ}^{*}:=\left\{g_{u}|g_{u}(\cdot )=[\Phi (\cdot ),u{]}_{\Omega },u\in \Omega \right\}$ *and with norm* ${\left\|g\right\|}_{{ℬ}^{*}}={\left\|u\right\|}_{\Omega }$*. The semi-inner product on the space* ${ℬ}^{*}$ *can be expressed as*

$${\left[g_{u}(\cdot ),g_{v}(\cdot )\right]}_{{ℬ}^{*}}:=[v,u{]}_{\Omega }$$ (20)

*And* ${ℬ}^{*}$ *is the conjugate space of* ℬ. ℬ *and* ${ℬ}^{*}$ *have the following bilinear form*

$${\left(f_{u}(\cdot ),g_{v}(\cdot )\right)}_{ℬ}:=[u,v{]}_{\Omega },\;u,v\in \Omega .$$ (21)

*A mapping G on* X×X *is a semi-inner-product reproducing kernel if and only if it satisfies the Eq (22),*

$$G(x,y)=[\Phi (x),\Phi (y){]}_{\Omega },\;x,y\in X$$ (22)

*where* Φ *is a function that maps X to a uniformly Fréchet differentiable and uniformly convex Banach space* Ω, *which satisfies Eq (18).*

**Proof.** Given that ℬ is a uniformly convex and uniformly Fréchet differentiable Banach space, we utilize the semi-inner product to explicitly characterize the dual bilinear mapping on $ℬ\times {ℬ}^{*}$. This construction satisfies all prerequisite assumptions for Theorem 2, particularly establishing the validity of the Riesz representation theorem in ℬ. Through the replacement of the bilinear mapping in Theorem 2 with the semi-inner product formulation (as specified in Eq (15)), we thereby complete the proof of the theorem.

**Fig 2:**
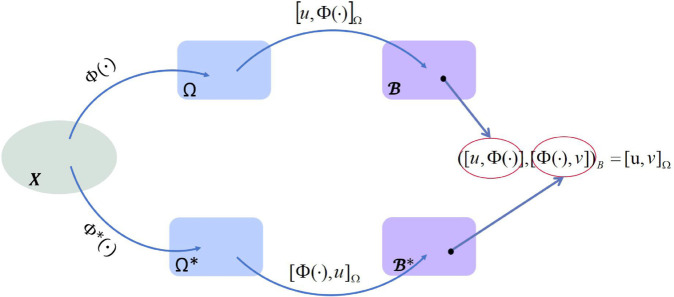
Schematic diagram of the reproducing kernel generation. ϕ(·), $\phi^{*}(\cdot )$ are bounded linear functionals on space *X*. Denote the space spanned by ϕ(X) as Ω, and the space spanned by $\phi^{*}(X)$ as $\Omega^{*}$. The relationship between the semi-inner product and the bilinear form given by Eq (15), we will use the mapping [u,ϕ(·)] and [ϕ(·),u] respectively on the spaces Ω and $\Omega^{*}$, ℬ and ${ℬ}^{*}$ are generated. The bilinear form on ℬ and ${ℬ}^{*}$ provides the expression of the semi-inner product reproducing kernel.

So far, by incorporating uniform Fréchet differentiable and uniform convex into the semi-inner product space, we have derived the theoretical form of the semi-inner product RKBS and obtained the corresponding expression of the reproducing kernel on the space. We now summarize the mapping relationships between different spaces as presented in Theorem 3. As shown in Fig 2, a schematic diagram illustrates the generation of the semi-inner product RKBS and the corresponding reproducing kernel is provided.

The theoretical foundation of RKBS has achieved substantial progress. The work by Xu [24] introduced a generalized RKBS framework by constructing left-sided and right-sided RKBS, extending the theory to asymmetric spaces. Song and Zhang [25] systematically investigated the theoretical mechanism of *l*^1^ norm based RKBS for improving algorithmic learning rates and provided rigorous theoretical proofs. However, existing studies remain confined to theoretical exploration, lacking explicit analytical forms of reproducing kernel functions and their integration into practical algorithms to address real-world problems.

To bridge the gap between theory and practical applications, this study aims to advance the practical implementation of RKBS in machine learning. Based on the algebraic properties of core computational operations in algorithms, we systematically analogize the structure of conventional inner products and adopt semi-inner products to replace inner products, thereby constructing a semi-inner-product reproducing kernel and its corresponding semi-inner-product RKBS theoretical framework. This framework not only preserves rigorous theoretical foundations but also enhances the applicability of reproducing kernel methods to real-world problems, offering a novel theoretical tool and practical pathway for complex data modeling. In the subsequent sections, we will first rigorously derive the mathematical construction of the semi-inner product reproducing kernel, then apply it to typical machine learning tasks through multiple empirical experiments, and systematically compare its performance with traditional kernel methods to validate the superiority and effectiveness of the proposed framework in practical applications.

## 4 Construction of semi-inner product reproducing Kernel

The construction of a semi-inner product reproducing kernel refers to defining a kernel function in a Banach space using a semi-inner product, which generalizes the concept of an inner product to spaces lacking symmetry or bilinearity. This construction allows the reproducing property, central in kernel methods, to extend beyond Hilbert spaces, enabling learning and approximation in more general Banach spaces.

Below, we present a reproducing kernel Banach space and a reproducing kernel on this space. Let X:=ℝ,S:=[−1,1], and *p*,*q* be conjugate numbers, p∈(1,+∞), $\frac{1}{p}\;+\;\frac{1}{q}=1$. Define $\Omega :=L^{p}(S),\Omega^{*}:=L^{q}(S)$ and mappings $\Phi :X\to \Omega ,\Phi^{*}:X\to \Omega^{*}$ by $\Phi (\omega ,t):=e^{-i\omega t},\;\Phi^{*}(\omega ,t):=e^{i\omega t},\;\omega \in X,t\in S$

For any $f\in L^{1}(\mathbb{R})$, the Fourier transform and its inverse are defined as the following two functions,


$$ℱ(t):=\int_{\mathbb{R}}f(\omega )e^{-i\omega t}\;d\omega ,\;\tilde{f}(t):=\int_{\mathbb{R}}f(\omega )e^{i\omega t}\;d\omega .$$

We construct two function spaces, ℬ and ${ℬ}^{*}$, generated by mapping ℱ and $\tilde{f}$, respectively:


$$ℬ:=\left\{f|\mathrm{supp}ℱ\subseteq S,ℱ\in L^{p}(S)\right\},\|f{\|}_{ℬ}:=\|ℱ{\|}_{L^{p}(S)}.$$

$${ℬ}^{*}:=\left\{g|\mathrm{supp}\tilde{g}\subseteq S,\tilde{g}\in L^{q}(S)\right\},\|g{\|}_{{ℬ}^{*}}:=\|\tilde{g}{\|}_{L^{q}(S)}.$$

For all f∈ℬ and $g\in {ℬ}^{*}$, the semi-inner product is given by $(f,g{)}_{ℬ}=\int_{\Omega }ℱ(t)\tilde{g}(t)dt.$ The reproducing kernel *K* on the space ℬ is defined by

$$K(x,y)={\left(\Phi (x),\Phi^{*}(y)\right)}_{\Omega }=\int_{S}e^{-ixt}e^{iyt}dt=\frac{2\sin(x-y)}{\left(x-y\right)}=2\mathrm{sinc}(x-y)$$ (23)

Following this construction, we introduce several discrete forms of semi-inner product reproducing kernels as shown in Eq (24), demonstrating the originality of this work.

Let *x* = {*x*_*i*_}, *y* = {*y*_*i*_}, i∈[1,n], we have

$$\left\{ \begin{array}{cc} K_{1}(x,y)= & \prod_{i=1}^{n}e^{x_{i}y_{i}}\\ K_{2}(x,y)= & \prod_{i=1}^{n}x_{i}y_{i}\\ K_{3}(x,y)= & e^{2\prod_{i=1}^{n}x_{i}y_{i}}\prod_{i=1}^{n}e^{x_{i}^{2}y_{i}^{2}}\\ K_{4}(x,y)= & \prod_{i=1}^{n}\frac{1}{1+x_{i}y_{i}}\\ K_{5}(x,y)= & \sum_{i=1}^{n}\frac{2}{i^{4}\pi^{4}}\sin(i\pi x_{i})\cos(i\pi y_{i})\\ K_{6}(x,y)= & \sum_{i=1}^{n}\frac{2}{i^{4}\pi^{4}}\sin(i\pi x_{i})\sin(i\pi y_{i})\\ K_{7}(x,y)= & \sum_{i=1}^{n}\frac{2}{i^{4}\pi^{4}}\cos(i\pi x_{i})\cos(i\pi y_{i})\\ K_{8}(x,y)= & \frac{1+\sum_{i=1}^{n}x_{i}^{p}y_{i}^{q}}{1+\sum_{i=1}^{n}x_{i}^{p}} \end{array}\right.$$ (24)

## 5 Experiment to verify the effectiveness of the semi-inner product Kernel function

### 5.1 Experimental setting

We validate the effectiveness of the proposed semi-inner product kernel function through four sets of experiments using different datasets. All datasets employed in our experiments were obtained from scikit-learn, an open-source Python machine learning library [26]. This library provides efficient tools for data mining and analysis and is widely adopted in both academic research and industry.

In the first experiment, we generated concentric circular test data comprising 800 sample points using the make_circles method from the scikit-learn library. This synthetic dataset was designed to evaluate the algorithm’s capability to correctly identify the optimal hyperplane separating the two distinct classes [26]. In the second experiment, we evaluated the algorithm’s classification performance using the classic Iris dataset [27]. In the third experiment, we employed the make_moons method from the scikit-learn library to generate a double semi-circle dataset, thereby further evaluating the algorithm’s classification capability on non-linearly separable data [26]. Finally, to systematically evaluate the comprehensive performance of the algorithm in handling multi-class classification problems, this study employs the standard wine dataset [28] for benchmark testing.

In all four experiments, we incorporated $K(x,y)=\prod_{i=1}^{n}e^{x_{i}y_{i}}$, the classical polynomial kernel, linear kernel, sigmoid kernel and the highly effective radial basis function(RBF) kernel as reproducing kernels in the support vector machine algorithm. Comparative experiments were conducted to validate the effectiveness of the semi-inner product kernel proposed in this study.

### 5.2 Experiment 1: Concentric circles

In the concentric circle experiment, each circle represents one class of data, totaling two classes. We aim to find a hyperplane that can separate the points of the two circles. Since the SVM algorithm seeks a hyperplane that separates the two classes of data while maximizing the margin between them, the optimal separating hyperplane in this experiment is a circular hyperplane located midway between the two concentric rings. We expect the algorithm to produce a separating hyperplane that lies midway between the two concentric circles.

**Fig 3:**
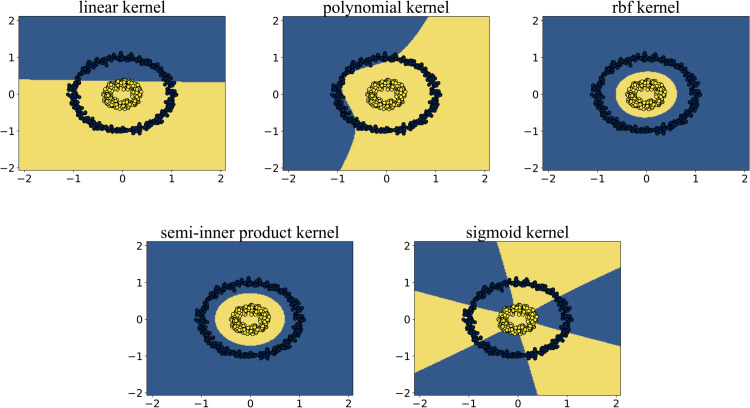
Results on the concentric circles dataset. Algorithms utilizing linear, polynomial, and sigmoid kernels failed to achieve correct classification due to their inability to capture the radial distribution characteristics of the data. In contrast, both the semi-inner-product kernel and RBF kernel successfully generated accurate decision boundaries, achieving perfect separation of the concentric structures.

As shown in Fig 3, the points in the blue region belong to one class of data, while those in the yellow region belong to the other class. The interface formed between the two colored regions is the separating hyperplane obtained by the algorithm. We can clearly observe that the KSVM algorithm based on the semi-inner product reproducing kernel and the RBF kernel successfully classifies the two classes of data points. Both algorithms identified a circular separating surface located midway between the two classes, achieving nearly identical results. In contrast, KSVM algorithms employing both the polynomial kernel and the linear kernel yielded similar results, failing to achieve correct classification between the two data categories. Likewise, the KSVM model based on the sigmoid kernel also demonstrated an inability to effectively separate the two classes.

### 5.3 Experiment 2: Iris dataset

**Fig 4:**
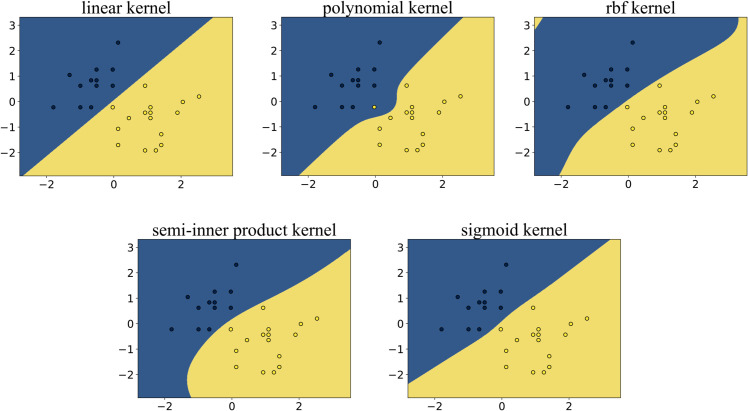
Experimental results obtained on the iris dataset. The polynomial kernel failed to yield satisfactory classification results. Although the semi-inner-product kernel, RBF kernel, sigmoid kernel, and linear kernel all achieved accurate data separation, the RBF kernel exhibited signs of overfitting.

In the classification experiment conducted on the Iris dataset, we classified two types of flowers. As shown in Fig 4, the blue points represent one class of data, while the yellow points represent the other class. The goal of the algorithm is to find the maximum-margin hyperplane that separates the two classes of data. The algorithms based on the five reproducing kernels each divided the data into two classes. The points in the blue region belong to one class, and those in the yellow region belong to the other class. The interface formed between the two colored regions is the separating hyperplane obtained by the algorithm.

From the figure, we can observe that:

- The KSVM algorithms based on the semi-inner-product reproducing kernel, RBF kernel, linear kernel, and sigmoid kernel all achieved perfect classification, successfully separating the two classes of data without any misclassified samples.
- In contrast, the KSVM algorithm employing the polynomial reproducing kernel exhibited noticeable misclassification, resulting in lower classification accuracy compared to the other methods.

### 5.4 Experiment 3: Double semi-circle

**Fig 5:**
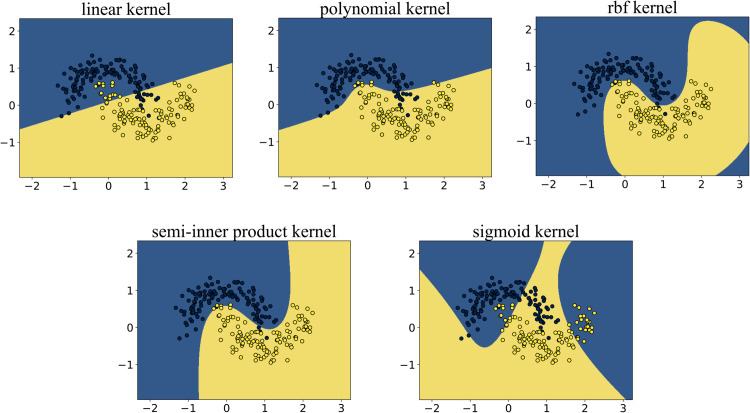
Experimental results obtained on the double semicircle dataset. The polynomial kernel did not obtain good classification results, while the semi-inner product kernel and RBF kernel both obtained accurate results.

In the experiment on the double semi-circle dataset, each semicircle represents one class of data, totaling two classes. As shown in Fig 5, the blue points belong to one class, while the yellow points belong to the other class. We aim to find a hyperplane that can separate the two classes of data. According to SVM theory, the optimal separating hyperplane in this case is a cubic curve located between the two classes of data points, and we expect the algorithm to produce this separating hyperplane. The algorithms based on the five reproducing kernels each divided the data into two classes. The points in the blue region belong to one class, and those in the yellow region belong to the other class. The interface formed between the two colored regions is the separating hyperplane obtained by the algorithm.

We can observe that:

- Polynomial and Linear Kernels: The separation hyperplanes generated by these kernels exhibit highly linear characteristics, resulting in insufficient model flexibility to capture nonlinear patterns in the data. Consequently, the classification performance is suboptimal, and the algorithm fails to effectively accomplish the given classification task.
- Sigmoid Kernel: Although this kernel introduces nonlinear mapping, the constructed hyperplane still fails to form an effective inter-class separation boundary, leading to significant misclassification phenomena.
- RBF and Semi-Inner-Product Reproducing Kernels: Despite minor localized classification errors, the hyperplanes constructed by these kernels effectively capture the essential distributional differences between the two classes, achieving high overall separation accuracy and successfully accomplishing the classification task.
- The separation hyperplane generated by the semi-inner-product reproducing kernel algorithm exhibits a cubic curve morphology, which most closely approximates the theoretically optimal classification interface among all compared models, without exhibiting overfitting. The geometric characteristics of this hyperplane reveal two key insights: First, the cubic curve structure effectively balances fitting accuracy and generalization capability; Second, its curvature variations align closely with the data distribution density, demonstrating the adaptive advantages of this kernel function in feature space mapping.

### 5.5 Experiment 4: Wine dataset

In this experiment, the wine dataset was selected as the benchmark platform [28]. This dataset comprises 178 wine samples, each represented by a 13-dimensional vector of continuous chemical features, including key physicochemical indicators such as alcohol content, malic acid concentration, alkalinity of ash, and total phenolic compounds. All samples originate from three distinct grape cultivars, forming a standard three-class supervised learning problem, thereby providing an ideal data foundation for validating the discriminative performance of complex classification algorithms. We trained and evaluated the algorithm by calculating the Accuracy, Precision, Recall, and F1-score for each kernel function on the test set, with the results summarized in Table 1.

**Table 1:** Comparison of classification performance among different kernel functions on the handwritten digits dataset (red indicates optimal values).

Kernel	Accuracy	Precision	Recall	F1-score
*RBF*	1.00	1.00	1.00	1.00
*poly*	0.97	0.96	0.98	0.97
*linear*	0.97	0.97	0.98	0.97
*sigmoid*	0.98	0.97	0.97	0.97
*semi-inner*	1.00	1.00	1.00	1.00

We can observe that:

- The RBF kernel achieved perfect scores of 1.00 across all four evaluation metrics, demonstrating its exceptional capability in modeling nonlinear feature mappings.
- The semi-inner-product reproducing kernel also attained optimal results of 1.00 on all metrics, matching the performance of the RBF kernel exactly.
- The polynomial and linear kernels exhibited comparable performance, both obtaining scores of 0.97 across all four metrics.
- The sigmoid kernel showed marginally better overall performance (0.98), though it still fell short of the optimal level.

Overall, the experimental results confirm the critical role of kernel function selection in determining classification performance. It is particularly noteworthy that the semi-inner-product reproducing kernel achieved classification performance fully comparable to the extensively validated RBF kernel, demonstrating significant potential for further generalization and optimization.

The integrated results from four experiments demonstrate that the separation hyperplanes constructed by KSVM algorithms using polynomial, linear, and sigmoid kernels exhibit significant limitations, resulting in generally suboptimal classification performance. In contrast, the semi-inner-product reproducing kernel and the RBF kernel achieve comparable classification accuracy in KSVM, with the semi-inner kernel demonstrating superior overfitting suppression on certain datasets. Notably, the semi-inner kernel effectively enhances nonlinear feature mapping capabilities through its reproducing properties, exhibiting exceptional generalization performance in complex boundary classification tasks. This study represents the first systematic experimental validation of the efficacy and application potential of the semi-inner-product reproducing kernel for nonlinear classification problems.

## 6 Conclusion and future work

In this study, we commence with a review of the conventional RKHS and its associated reproducing kernels. We define the necessary and sufficient conditions for the existence of a reproducing kernel and examine the construction methodologies of RKHS. Finally, we consolidate the core theorems and conditions relevant to the proof of RKHS, introduce the analogous definition of RKBS, and present the necessary and sufficient conditions for the existence of a reproducing kernel in RKBS, supported by a comprehensive proof process.

We address the existing issues in RKBS by integrating a semi-inner product into the space and augmenting it with two additional properties, ultimately establishing a semi-inner product RKBS that satisfies the required conditions. This demonstrates the originality of this study. Subsequently, we provide an example demonstrating the generation of a reproducing kernel and the construction process of each space. To facilitate the application of the semi-inner product reproducing kernel in machine learning algorithms, we introduce a discrete form of the reproducing kernel based on the example. Through systematic comparative experiments, we have not only validated the effectiveness of the semi-inner-product reproducing kernel in algorithmic design, but also demonstrated its significantly superior classification performance over Sigmoid, polynomial, and linear kernels, while achieving results comparable to those of the RBF kernel. These empirical findings collectively indicate that the proposed kernel function maintains theoretical innovativeness while possessing substantial value for practical application and dissemination.

However, there are still several unresolved issues in this work that require further improvement in future research. First, we need to provide theoretical proof based on RKBS for various algorithms. Second, this work did not systematically perform a large number of evaluations but only validated its effectiveness. Additionally, RKHS-based algorithms have a broad range of application scenarios. We need to identify application scenarios where RKBS-based algorithms offer advantages and explain why they outperform in these scenarios. These research issues will be addressed in future work.

We intend to leverage the semi-inner product reproducing kernel to enhance machine learning algorithms, such as kernel method-based algorithms, support vector machines, and principal component analysis, to develop a more comprehensive theory of machine learning algorithms grounded in the framework of reproducing kernel Banach space. Through a motivating example, we not only verified the effectiveness of the semi-inner-product reproducing kernel in the algorithm but also achieved superior results compared to the polynomial reproducing kernel. The results obtained from the KSVM using the semi-inner-product reproducing kernel are identical to those obtained from the KSVM with the RBF kernel, demonstrating the practical utility of the semi-inner-product reproducing kernel.

The kernel method originated in the early 20th century after Mercer’s theorem was proposed and Hilbert space was established. Since then, primary research in kernel methods has focused on algorithm development and application practices within the framework of RKHS. We hope this paper will attract machine learning researchers to the theoretical and algorithmic development in RKBS, enriching the study of kernel methods and marking a milestone in the history of kernel method research.
